# Genome Sequences of *Beet curly top Iran virus*, *Oat dwarf virus*, *Turnip curly top virus*, and *Wheat dwarf virus* Identified in Leafhoppers

**DOI:** 10.1128/genomeA.01674-16

**Published:** 2017-02-23

**Authors:** Mehdi Kamali, Jahangir Heydarnejad, Najmeh Pouramini, Hossain Masumi, Kata Farkas, Simona Kraberger, Arvind Varsani

**Affiliations:** a Department of Plant Protection, College of Agriculture, Shahid Bahonar University of Kerman, Kerman, Iran; b School of Biological Sciences, University of Canterbury, Christchurch, New Zealand; c Department of Clinical Laboratory Sciences, University of Cape Town, Cape Town, South Africa; d Department of Microbiology, Immunology and Pathology, Colorado State University, Fort Collins, Colorado, USA; e The Biodesign Center for Fundamental and Applied Microbiomics, Center for Evolution and Medicine, School of Life Sciences, Arizona State University, Tempe, Arizona, USA

## Abstract

Implementation of a vector-enabled metagenomics approach resulted in the identification of various geminiviruses. We identified the genome sequences of *Beet curly top Iran virus*, *Turnip curly top viruses*, *Oat dwarf viruses*, the first from Iran, and *Wheat dwarf virus* from leafhoppers feeding on beet, parsley, pumpkin, and turnip plants.

## GENOME ANNOUNCEMENT

New molecular-based methods have facilitated the rapid identification of various plant and animal viruses, especially small single-stranded DNA viruses. Furthermore, vector-enabled metagenomic (VEM) approaches using these methods have proven very useful for the identification of plant virus circulation in various agricultural regions (1–4). Here, we used a VEM approach to identify geminiviruses (5) circulating in farms in Zafar-Abad, Iran, in 2013 by sampling of plant leafhoppers from different genera feeding on beet (*Beta vulgaris*; *n* = 1), parsley (*Petroselinum crispum*; *n* = 1), pumpkin (*Cucurbita pepo*; *n* = 1), and turnip (*Brassica rapa*; *n* = 3) plants. The insects were homogenized in SM buffer and processed as described by Dayaram et al. (6–9). The viral DNA was enriched for circular molecules using rolling-circle amplification, and this was sequenced on an Illumina sequencing platform at Novogene (Hong Kong). The paired-end reads were *de novo* assembled using ABySS 1.9 (10), and viral sequences were identified using BLASTx against a local viral protein sequence database. Contigs with BLASTx hits to geminivirus sequences (5, 11) were identified. Abutting primers were designed and used for PCR amplification to recover full viral genomes. These were cloned and sequenced at Macrogen, Inc. (South Korea). Two *Oat dwarf virus* (ODV) genomes that were recovered from leafhoppers feeding on beet and turnip share 99.8% identity and 97% with the only other ODV identified infecting oat (*Avena sativa*) in Germany. To date, ODV has not been reported in Iran, and therefore, these genome sequences (accession numbers KX533458 to KX533459) represent the first genome sequences of ODV from Iran. Six *Wheat dwarf virus* (WDV) genomes recovered from leafhoppers feeding on beet (*n* = 2), turnip (*n* = 2), pumpkin (*n* = 1), and parsley (*n* = 1) display 13.6% diversity (accession numbers KX533460 to KX533465); however, compared with genomes of WDV in public databases, they are most closely related to recently identified sequences from Iran, sharing >97% genome-wide identity. The *Beet curly top Iran virus* (BCTIV; accession number KX533466) and *Turnip curly top virus* (TCTV; accession numbers KX533467 to KX533468) sequences share 94% and 99% identity with previously described isolates, respectively. The BCTIV and TCTV genomes were recovered from leafhoppers feeding on turnip, and the two TCTV genomes share 90% identity.

Leafhoppers transmitted several destructive viral and prokaryotic diseases, such as curly top of sugar beet and turnip crops (12) and citrus stubborn (13) in Iran. We analyzed the beet, parsley, pumpkin, and turnip plants being fed on by leafhoppers in this study and did not detect ODV or WDV infection. This is perhaps not surprising, as vectors play a crucial role in viral transmission and movement, and *Psammotettix alienus* has been shown to transmit ODV (14) and WDV (15). It is therefore probable that these leafhoppers acquired ODV and WDV when feeding on infected grasses prior to being sampled.

VEM has proven a useful tool not only in this case where we identified and recovered the genomes of ODVs and WDVs circulating in Fars province of Iran but also in identifying the first mastrevirus in the Caribbean (2), novel begomovirus species (3), and known and novel plant-infecting viruses in an unlikely vector, mosquitoes (16).

### Accession number(s).

The complete genome sequences for the four viruses have been deposited in GenBank under accession numbers KX533466 (BCTIV), KX533458 to KX533459 (ODV), KX533467 to KX533468 (TCTV), and KX533460 to KX533465 (WDV).

## References

[1] Rosario K, Marr C, Varsani A, Kraberger S, Stainton D, Moriones E, Polston JE, Breitbart M 2016 Begomovirus-associated satellite DNA diversity captured through vector-enabled metagenomic (VEM) surveys using whiteflies (Aleyrodidae). Viruses 8:e36. doi:10.3390/v8020036. 26848679 PMC4776191

[2] Rosario K, Padilla-Rodriguez M, Kraberger S, Stainton D, Martin DP, Breitbart M, Varsani A 2013 Discovery of a novel mastrevirus and alphasatellite-like circular DNA in dragonflies (Epiprocta) from Puerto Rico. Virus Res 171:231–237. doi:10.1016/j.virusres.2012.10.017. 23116593

[3] Rosario K, Seah YM, Marr C, Varsani A, Kraberger S, Stainton D, Moriones E, Polston JE, Duffy S, Breitbart M 2015 Vector-enabled metagenomic (VEM) surveys using whiteflies (Aleyrodidae) reveal novel *Begomovirus* species in the new and old worlds. Viruses 7:5553–5570. doi:10.3390/v7102895. 26516898 PMC4632403

[4] Ng TF, Duffy S, Polston JE, Bixby E, Vallad GE, Breitbart M 2011 Exploring the diversity of plant DNA viruses and their satellites using vector-enabled metagenomics on whiteflies. PLoS One 6:e19050. doi:10.1371/journal.pone.0019050. 21544196 PMC3081322

[5] Varsani A, Navas-Castillo J, Moriones E, Hernández-Zepeda C, Idris A, Brown JK, Murilo Zerbini F, Martin DP 2014 Establishment of three new genera in the family *Geminiviridae*: *Becurtovirus*, *Eragrovirus* and *Turncurtovirus*. Arch Virol 159:2193–2203. doi:10.1007/s00705-014-2050-2. 24658781

[6] Dayaram A, Galatowitsch M, Harding JS, Argüello-Astorga GR, Varsani A 2014 Novel circular DNA viruses identified in *Procordulia grayi* and *Xanthocnemis zealandica* larvae using metagenomic approaches. Infect Genet Evol 22:134–141. doi:10.1016/j.meegid.2014.01.013. 24462907

[7] Dayaram A, Galatowitsch ML, Argüello-Astorga GR, van Bysterveldt K, Kraberger S, Stainton D, Harding JS, Roumagnac P, Martin DP, Lefeuvre P, Varsani A 2016 Diverse circular replication-associated protein encoding viruses circulating in invertebrates within a lake ecosystem. Infect Genet Evol 39:304–316. doi:10.1016/j.meegid.2016.02.011. 26873065

[8] Dayaram A, Potter KA, Moline AB, Rosenstein DD, Marinov M, Thomas JE, Breitbart M, Rosario K, Argüello-Astorga GR, Varsani A 2013 High global diversity of cycloviruses amongst dragonflies. J Gen Virol 94:1827–1840. doi:10.1099/vir.0.052654-0. 23596268

[9] Dayaram A, Potter KA, Pailes R, Marinov M, Rosenstein DD, Varsani A 2015 Identification of diverse circular single-stranded DNA viruses in adult dragonflies and damselflies (Insecta: Odonata) of Arizona and Oklahoma, USA. Infect Genet Evol 30:278–287. doi:10.1016/j.meegid.2014.12.037. 25577985

[10] Simpson JT, Wong K, Jackman SD, Schein JE, Jones SJ, Birol I 2009 ABySS: a parallel assembler for short read sequence data. Genome Res 19:1117–1123. doi:10.1101/gr.089532.108. 19251739 PMC2694472

[11] Muhire B, Martin DP, Brown JK, Navas-Castillo J, Moriones E, Zerbini FM, Rivera-Bustamante R, Malathi VG, Briddon RW, Varsani A 2013 A genome-wide pairwise-identity-based proposal for the classification of viruses in the genus *Mastrevirus* (family *Geminiviridae*). Arch Virol 158:1411–1424. doi:10.1007/s00705-012-1601-7. 23340592

[12] Hosseini Abhari E, Heydarnejad J, Massumi H, Hosseini Pour A, Izadpanah K 2016 Natural hosts, vector and molecular detection of *Beet curly top virus* (BCTV) in southeast of Iran. Proc 2nd Asian Plant Pathol Cong, Singapore.

[13] Omidi M, Hosseini-Pour A, Rahimian H, Massumi H, Saillard C 2011 Identification of *Circulifer haematoceps* (Hemiptera:Cicadellidae) as vector of *Spiroplasma citri* in the Kerman Province of Iran. J Plant Pathol 93:167–172.

[14] Schubert J, Habekuss A, Kazmaier K, Jeske H 2007 Surveying cereal-infecting geminiviruses in Germany—diagnostics and direct sequencing using rolling circle amplification. Virus Res 127:61–70. doi:10.1016/j.virusres.2007.03.018. 17449126

[15] Lotfipour M, Behjatnia SAA, Afsharifar A, Izadpanah K 2013 Surveying cereal-infecting geminiviruses in Germany—diagnostics and direct sequencing using rolling circle amplification. Iran J Plant Pathol 49:17–31.

[16] Ng TF, Willner DL, Lim YW, Schmieder R, Chau B, Nilsson C, Anthony S, Ruan Y, Rohwer F, Breitbart M 2011 Broad surveys of DNA viral diversity obtained through viral metagenomics of mosquitoes. PLoS One 6:e20579. doi:10.1371/journal.pone.0020579. 21674005 PMC3108952

